# Cross‐Linked Double Network Graphene Oxide/Polymer Composites for Efficient Coagulation‐Flocculation

**DOI:** 10.1002/gch2.201900051

**Published:** 2019-10-01

**Authors:** Shuyuan Lin, Qilong Li, Yujia Zhong, Jing Li, Xuanliang Zhao, Min Wang, Guoke Zhao, Jialiang Pan, Hongwei Zhu

**Affiliations:** ^1^ State Key Laboratory of New Ceramics and Fine Processing School of Materials Science and Engineering Tsinghua University Beijing 100084 China; ^2^ Fangda Carbon New Material Co., Ltd. Lanzhou 730084 Gansu China

**Keywords:** coagulation, composites, flocculation, graphene oxide

## Abstract

Hybrid coagulant/flocculant consisting of nanomaterials have tremendous potential in solid‐liquid separation and can be applied to the coagulation‐flocculation‐sedimentation process of water treatment. In this work, inspired by the mineralization in nature, a graphene oxide/polymer‐based hybrid coagulant/flocculant that precipitates large‐scale, multicomponent (e.g., dyes, heavy metal ions, and nanoparticles) and complex pollutants simultaneously at room temperature by forming double‐network hydrogel through bioinspired Ca^2+^ crosslinking, is developed for the purification of wastewater. The coagulation‐flocculation‐sedimentation method developed here also provides a novel strategy for the preparation of macroscopic assemblies of multicomponents that can be applied to various application fields.

## Graphical abstract

This work demonstrates a graphene oxide/polymer‐based hybrid coagulant/flocculant, precipitating large‐scale, multicomponent (e.g., dyes, heavy metal ions, and nanoparticles) and complex pollutants simultaneously at room temperature by forming a double‐network hydrogel through Ca^2+^ crosslinking to achieve the purification of wastewater. This method also provides a novel strategy for the preparation of macroscopic assemblies of multicomponents that can be applied to various application fields.

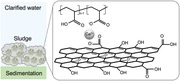

## 1 Introduction

Hybrid/composite materials have emerged as new materials that pose tremendous potentials in solid‐liquid separation because of their superior performance compared with those of conventional environmental materials.^1^, ^2^ Recently, the application of nanomaterials (such as carbon nanotubes and graphene‐based materials) in water treatment and environmental remediation has attracted considerable attention owing to their large surface area, tunable functionalities, and unique physical and chemical properties.^3^, ^4^, ^5^, ^6^

For example, solid‐liquid separation through coagulation‐flocculation is an important unit operation in water treatment and for sludge dewatering in pulp and paper processing, pharmaceuticals, cosmetics, food, mineral processing, and so forth.^7^, ^8^, ^9^ The continuous increase of industry needs for efficient and effective materials in water treatment has spurred the development of hybrid materials for coagulation‐flocculation of waste water.^10^, ^11^, ^12^ Various materials have been developed for coagulation and flocculation purposes, including inorganic‐based coagulants, organic‐based flocculants, and hybrid materials.^13^, ^14^, ^15^, ^16^ Although a wide range of materials has been successfully used in removing pollutants from wastewater, there is still a need to improve their performance. One of the major issues related to the coagulation‐flocculation process is the toxicity and health hazard possessed by inorganic coagulants. On the other hand, the use of natural coagulants is not effective in removing chemical oxygen demand (COD) due to their organic properties. In addition, the coagulation‐flocculation process involving natural coagulants was also found to be less effective in treating wastewater containing heavy metals or emerging contaminants.

**Figure 1:**
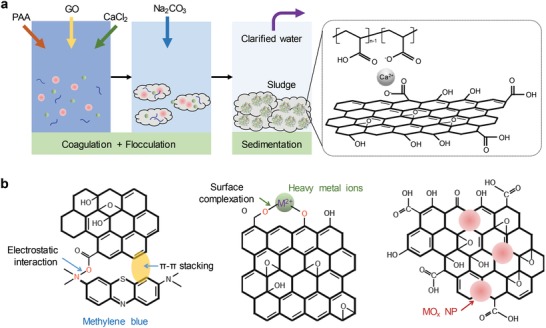
a) The scheme and mechanism of the coagulation‐flocculation‐sedimentation process. b) The mechanism of the interaction of GO with dyes, heavy metal ions, and nanoparticles.

In this study, we introduced a graphene oxide (GO)/polymer‐based hybrid coagulant/flocculant by forming double‐network hydrogel through bioinspired Ca^2+^ crosslinking during the coagulation‐flocculation process (**Figure** **1**a).^17^ When applied to water treatment, GO/polymer‐based hybrid coagulant could precipitate multiple pollutants (e.g., dyes, heavy metal ions, and nanoparticles) simultaneously at room temperature to achieve the purification of wastewater. This method is facile and environmentally friend without involving any toxic cross‐linker. Meanwhile, it also provides a new strategy for the preparation of macroscopic assemblies containing multicomponent.

## 2 Results and Discussion

Oxygen atoms on GO in the forms of epoxy, hydroxyl, and carboxyl groups can strongly interact with positively charged molecules primarily because of their strong electrostatic interactions.^18^, ^19^ As shown in Figure 1b, the interactions between dye molecules and GO include: (i) electrostatic interaction between GO and cationic dye, and (ii) π–π stacking interaction between aromatic moiety of dye and the delocalized π‐electron system of GO. In the present study, methylene blue (MB), one of the frequently used organic dyes, was chosen as a model dye to investigate the performance of coagulation‐flocculation‐sedimentation approach for removal of organic dyes from aqueous solution.

**Figure 2:**
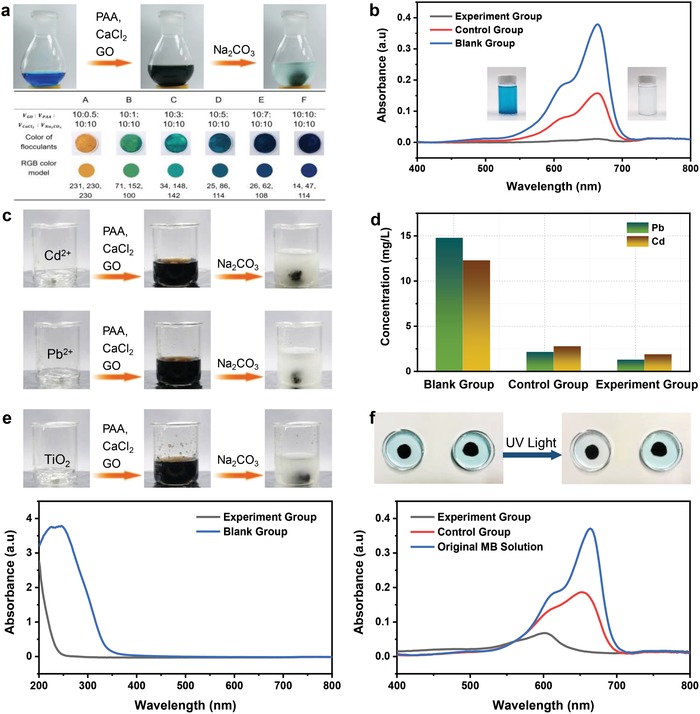
a) Schematic diagram of MB removal experiment in the experiment group (top panel) and colors of precipitates with different compositions (bottom panel). b) UV–vis spectra of the solution after reaction in the experiment group, the control group, and the blank group. c) Removal of Cd^2+^ and Pb^2+^ in the experiment groups. d) The content of Pb and Cd in the solutions after reaction measured by ICP‐AES. e) The coagulation‐flocculation‐precipitation experiment of TiO_2_ for the experiment group (top panel) and UV–vis spectra of the solution after reaction (bottom panel); f) contrast experiments of photocatalytic degradation of MB: the results of the experiment group and the control group (top panel); UV–vis spectra of original MB solution and the solutions after reaction (bottom panel).

**Figure** **2**a (top panel) and Figure S1 (Supporting Information) show the comparative experiments on precipitation of MB with and without involving GO. The parameters of the experiment group, the control group, and the blank group in the comparative experiments on the precipitation of MB are shown in Table S1 (Supporting Information), and the entire experiment process is illustrated in Movie S1 (Supporting Information). In the experiment group, poly(acrylic acid) (PAA), CaCl_2_, and GO solution were first mixed with MB solution under stirring, followed by the slow addition of Na_2_CO_3_ solution. The final solution turned to clear, and a fluffy green precipitate was generated very quickly (<1 min) (Figure 2a, top panel). In the control group, the same volume of deionized (DI) water instead of GO solution was added to MB solution together with PAA and CaCl_2_ solution under stirring. After the Na_2_CO_3_ solution was added slowly, a small amount of blue viscous precipitate was obtained, but the solution remained blue (Figure S1, Supporting Information). The collected solutions after removal of the precipitate by simply decantation were characterized with the UV–vis absorption spectra to estimate the residual MB concentration and removal efficiency. The original MB aqueous solution was blue. After the coagulation‐flocculation‐sedimentation experiment, the color of the collected solution in the experiment group faded, which was different from those of the control group and the blank group, suggesting that GO facilitated the precipitation of dissolved dyes.

The UV–vis absorption spectra further confirmed this observation. Figure 2b shows the UV–vis absorption spectra of the experiment group, the control group, and the blank group. The absorption intensity of characteristic MB peak at 664 nm in the experiment group decreased significantly compared with those of the control group and the blank group, indicating the removal of MB under the help of GO. Since dyes usually have complex aromatic molecular structures, they are stable and difficult to be removed by traditional wastewater treatment technology. Our method provided a one‐step, facile, and low‐energy‐consuming protocol for dye removal, which has promising potential for applications in sewage treatment.

Interestingly, in addition to removing dyes from the solution, our method has the advantage of producing precipitates with added value. As shown in Figure 2a (bottom panel), by adjusting the reactant ratio of GO and MB, precipitates with different colors could be obtained. The formed colorful precipitates were stretchable, shapeable, self‐healable, and easy to be processed in the wet state, but they were tough and rigid in the dried state, which shows the advantage of recyclability and can be used to form various complex architectures.

GO interacts with heavy metal ions through surface complexation (Figure 1b).^20^, ^21^, ^22^ Figure 2c and Figure S2 (Supporting Information) show the coagulation‐flocculation‐sedimentation experiments on the precipitation of Cd^2+^ and Pb^2+^ with and without GO participation, respectively. The parameters of the experiment group, the control group, and the blank group in the entire comparative experiments on the precipitation of heavy metal ions are listed in Table S2 (Supporting Information). As shown in Figure 2c, in the experiment groups, the addition of Na_2_CO_3_ solution to a solution consisting of PAA, CaCl_2_, GO, and heavy metal ions generated a fluffy brown precipitate. In contrast, in the control groups, the addition of Na_2_CO_3_ solution to a mixed solution of PAA, CaCl_2_, and the heavy metal ions only formed a small amount of white viscous precipitates (Figure S2, Supporting Information). After precipitates were removed, the amount of heavy metal ions remained in the reactor was measured by inductively coupled plasma‐atomic emission spectrometry (ICP‐AES), and the effect of GO on the removal of heavy metal ions is shown in Figure 2d. When GO participated in the reaction, the concentrations of Pb^2+^ and Cd^2+^ were decreased by 91.2 and 84.6%, respectively, compared with the blank group. However, without the adsorption of heavy metal ions by GO, the concentrations of Pb^2+^ and Cd^2+^ were decreased by 85.4 and 77.3%, respectively, compared with that of the blank group. Therefore, the coagulation‐flocculation‐sedimentation method developed in this study offered a fast and efficient approach to remove heavy metal ions, which has promising potential for applications in the water purification field.

Nanoparticles can interact with GO sheets through physisorption, electrostatic binding, and charge transfer interactions (Figure 1b).^23^, ^24^ The composite of GO and metal oxide nanoparticles play important roles, such as photocatalysts, adsorbents, and disinfectants in water treatment, which is effective against water pollutants. Since TiO_2_ is one of the metal oxides that frequently used as photocatalysts, it has been widely explored for water purification.^25^, ^26^, ^27^, ^28^ Herein, a GO/TiO_2_ composite consisting of GO and TiO_2_ has been successfully synthesized by a coagulation‐flocculation‐sedimentation method in the present study. Figure 2e (top panel) shows the process for the preparation of GO/TiO_2_ composite. Table S3 (Supporting Information) shows the parameters of the experiment group and the blank group in the entire comparative experiments on the precipitation of TiO_2_. After the reaction, the content of residual TiO_2_ in the experiment group and the blank group were measured by UV–vis spectroscopy (Figure 2e, bottom panel). The intensity of characteristic TiO_2_ absorption peak in the experiment group was much weaker than that in the blank group, indicating that the TiO_2_ concentration of the residual solution in the experiment group was lower than that of the blank group. It has been reported that MB in water can be photocatalytically degraded by TiO_2_ under UV light irradiation.^29^ To further confirm the existence of TiO_2_ involved in the precipitate of the experiment group, the precipitate was placed in a petri dish containing MB solution for photocatalytic degradation. For comparison, another precipitate without TiO_2_ was prepared and placed in another identical petri dish with MB solution (Figure 2f, top panel). The parameters of the experiment group and the control group of the above comparative experiments on the removal of MB by precipitates are shown in Table S4 (Supporting Information). As shown in Figure 2f (top panel), under UV light irradiation for about 15 min, the solution color of the experiment group (left) was faded, whereas that of the control group (right) retained. As shown in Figure 2f (bottom panel), the UV–vis absorption spectra confirmed the content of MB almost before and after reaction of the experiment group and the control group. Because of the adsorption of MB by GO, the characteristic peak intensity of MB in the control group decreased comparing with that of original MB solution.^30^ In the experiment group, the peak intensity decreased more due to the photocatalytic degradation by TiO_2_ besides GO adsorption, indicating the existence of TiO_2_ in the precipitate of the experiment group.^31^, ^32^ In other words, assembled macrocomposite containing GO and TiO_2_ was successfully synthesized by the coagulation‐flocculation‐sedimentation process.

**Figure 3:**
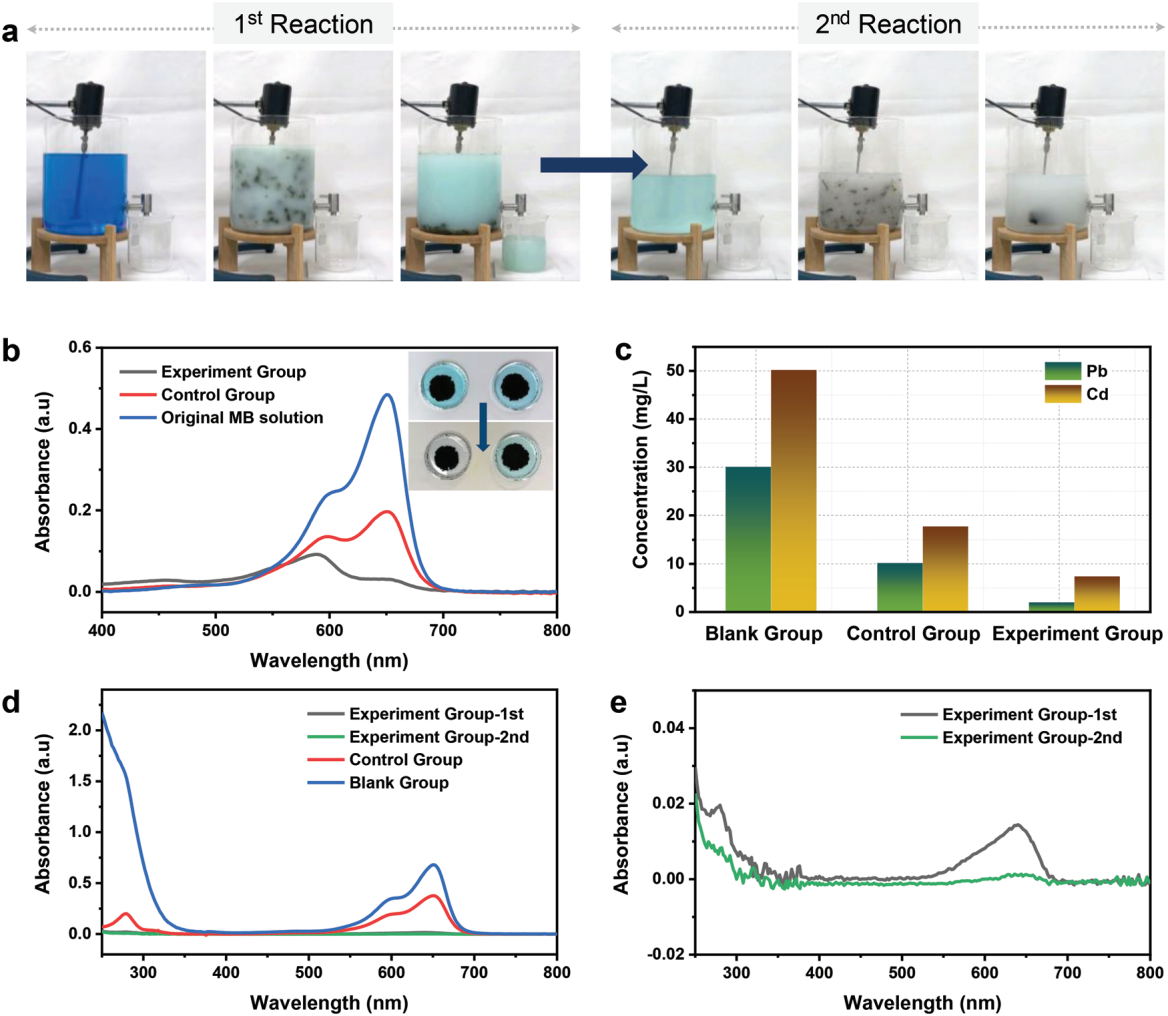
a) Screenshots of first and second reaction processes in large‐scale purification experiments of multicomponent solution of the experiment groups. b) UV–vis spectra of original MB solution and the solutions in the experiment group and the control group after photocatalytic degradation of MB. Contrast experiments of photocatalytic degradation of MB of the experiment group and the control group (inset). c) The content of Pb and Cd in the solution of the blank group, the control group, and the experiment group (first reaction) after reaction measured by ICP‐AES. d) UV–vis spectra of the experiment groups, the control group, and the blank group. e) UV–vis spectra of the solution after first and second reaction in the experiment groups (enlarged view of (d)).

It was noteworthy that the coagulation‐flocculation‐sedimentation approach consisting of GO, PAA, CaCl_2_, and Na_2_CO_3_ can be applied to large‐scale and multicomponent complex solutions to achieve the purification of residual solution and precipitation of various components. **Figure** **3**a and Figure S3 (Supporting Information) show the comparative experiments on the purification of a multicomponent solution with and without GO participation. The parameters of the experiment group, the control group, and the blank group in the comparative experiments are shown in Table S5 (Supporting Information). Evidently, as shown in Figure 3a, Figure S3 and Movie S2 (Supporting Information), the solution with the addition of GO faded while the solution without GO participation remained blue. It was worth mentioning that after the first reaction, the reaction solution was slightly green, indicating that GO was insufficient and components were not completely removed during the reaction (Figure 3a (left panel) and Movie S3, Supporting Information). The second reaction is to add a small amount of GO, PAA, CaCl_2_, and Na_2_CO_3_ into 4 L of the filtrate after the first reaction. After the second reaction, the filtrate was completely decolorized, indicating that the removal of components was basically completed (Figure 3a (right panel) and Movie S4, Supporting Information). After standing, the decolorized filtrate became clarified (Figure S4, Supporting Information). The presence of TiO_2_ in the precipitate of the first reaction in Figure 3a was determined in Figure 3b according to the photocatalytic properties of TiO_2_. Comparative experiments on removal of MB by participates are shown in Figure 3b (inset) and parameters of the control group of the above comparative experiments are shown in Table S6 (Supporting Information).

As shown in the top panel of Figure 3b (inset), the precipitates of the experiment group (left) and the control group (right) were added to the MB solution. The bottom panel of Figure 3b (inset) shows the solution in the petri dish of the experiment group quickly faded while that in the petri dish of the control group retained. Figure 3b shows the UV–vis absorption spectra of the solution before and after the photocatalytic reaction. It can be seen that after the reaction, the absorption peak of MB in the solution of the experiment group and the control group both decreased compared with the original MB solution, and the absorption peak of MB in the experiment group was much lower than that in the control group, which further confirmed the existence of TiO_2_ in the precipitate of the experiment group.

Furthermore, the removal efficiencies of heavy metal ions in comparative experiments are analyzed in Figure 3c. The concentrations of heavy metal ions in the residual solutions of the experiment group, the control group, and the blank group were determined by ICP‐AES. ICP results showed that the heavy metal ion concentration of the residual solution in the experiment group was the lowest, indicating that the removal of heavy metal ions was achieved through the coagulation‐flocculation‐sedimentation process with the participation of GO. The UV–vis absorption spectra in Figure 3d show that the TiO_2_ and MB characteristic absorption peak of the residual solution in the experiment groups were the weakest, indicating the TiO_2_ and MB concentration in the residual solution of the experiment group were the lowest. Figure 3e enlarges the results of two reactions in the experiment groups in Figure 3d. The characteristic absorption peaks of the experiment groups after the second reaction are significantly lower than those of the first reaction, indicating that it is possible to gradually reduce the content of components in the residual liquid by multiple reactions and ultimately achieve the purpose of purification.

## 3 Conclusion

In summary, the coagulation‐flocculation‐sedimentation method developed in this study has advantages of high adaptability, practicability, energy‐saving, and speediness, which is expected to be applied to multicomponent, large‐scale, and complex aqueous solution. When applied to water treatment, GO/polymer‐based hybrid coagulant precipitated multiple pollutants simultaneously at room temperature to achieve the purification of wastewater. Moreover, the coagulation‐flocculation‐sedimentation method developed in this work provides a novel strategy for the preparation of macroscopic assemblies of multicomponents to expand the application scope of this developed protocol and to meet a variety of specific requirements in various application fields, such as energy‐storage electrodes, actuators, and sensors.

## 4 Experimental Section

*Materials and Chemicals*: Natural graphite was purchased from Qingdao Tengshengda Graphite Co., Ltd. CaCl_2_ and Na_2_CO_3_ were purchased from General‐Reagent, Titan Scientific Co., Ltd. PAA was purchased from Sigma‐Aldrich Co., Ltd. Methylene blue, PbCl_2_, and CdCl_2_ were purchased from Titan Scientific Co., Ltd. TiO_2_ was purchased from Aladdin Co., Ltd. Concentrated sulfuric acid (H_2_SO_4_, 98%), potassium permanganate (KMnO_4_), sodium nitrate (NaNO_3_), hydrogen peroxide (H_2_O_2_, 30%), and hydrochloric acid (HCl) were purchased from Beijing Sinopharm Chemical Reagent Co., Ltd.

*Preparation of GO*: GO was prepared from natural graphite by a modified Hummers method using H_2_SO_4_, KMnO_4_, and NaNO_3_ for oxidation. GO nanosheets were obtained after sonication and drying, and subsequently prepared into 1 mg mL^−1^ GO solution.

*Characterizations*: UV–vis absorption spectra were conducted using a UV–vis spectrophotometer (Hitach UV2800). The concentrations of heavy metals (Cd, Pb) were analyzed by ICP‐AES technique (IRIS Intrepid II).

## Conflict of Interest

The authors declare no conflict of interest.

## Supporting information

Supplementary

Supplementary

Supplementary

Supplementary

Supplementary
