# Supplementary material for: Cross‐Linked Double Network Graphene Oxide/Polymer Composites for Efficient Coagulation‐Flocculation
Source: Glob Chall. 2019 Oct 1;4(1):1900051. doi: 10.1002/gch2.201900051 (PMC6957014; doi:10.1002/gch2.201900051)
Supplement: Supplementary file 1 — Supplementary [file GCH2-4-1900051-s002.pdf]

Copyright WILEY-VCH Verlag GmbH & Co. KGaA, 69469 Weinheim, Germany, 2019.

Global Challenges
Open Access

Supporting Information

for *Global Challenges,* DOI: 10.1002/gch2.201900051

Cross-Linked Double Network Graphene Oxide/Polymer Composites for Efficient Coagulation-Flocculation

*Shuyuan Lin, Qilong Li, Yujia Zhong, Jing Li, Xuanliang Zhao, Min Wang, Guoke Zhao, Jialiang Pan, and Hongwei Zhu**

Supporting Information

Cross-linked Double Network Graphene Oxide/Polymer Composites for Efficient Coagulation-Flocculation

Shuyuan Lin[a], *Qilong Li*[b], *Yujia Zhong*[a], *Jing Li*[a], *Xuanliang Zhao*[a], *Min Wang*[a], *Guoke Zhao*[a], *Jialiang Pan*[a], *Hongwei Zhu**[a]

[a]State Key Laboratory of New Ceramics and Fine Processing, School of Materials Science and Engineering, Tsinghua University, Beijing 100084, China
[b]Fangda Carbon New Material Co., Ltd., Lanzhou 730084, Gansu, China

This file includes:

Figure S1. Precipitation of MB without GO.

Figure S2. Precipitation of Cd^{2+} and Pb^{2+} without GO.

Figure S3. Purification of a multi-component solution without GO.

Figure S4. The filtrate before and after standing.

Table S1. Comparative experiments on precipitation of MB.

Table S2. Comparative experiments on precipitation of heavy metal ions.

Table S3. Comparative experiments on precipitation of TiO_2.

Table S4. Comparative experiments on the removal of MB by precipitation.

Table S5. Comparative experiments on purification of large-scale, multi-component complex solutions.

Table S6. Comparative experiments of control group on the removal of MB by precipitation.

Movie S1. Comparative experiments on precipitation of MB with and without GO.

Movie S2. Purification of a multi-component solution without GO.

Movie S3. 1st purification of a multi-component solution with GO.

Movie S4. 2nd purification of a multi-component solution with GO.

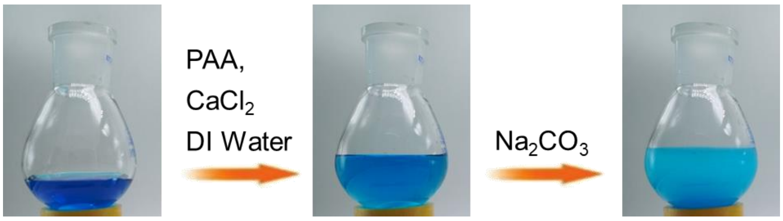

Figure S1. Precipitation of MB without GO.

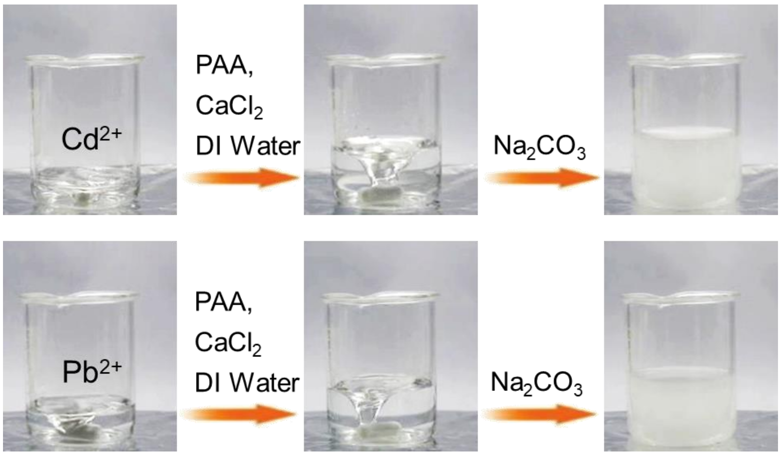

Figure S2. Precipitation of Cd^{2+} and Pb^{2+} without GO.

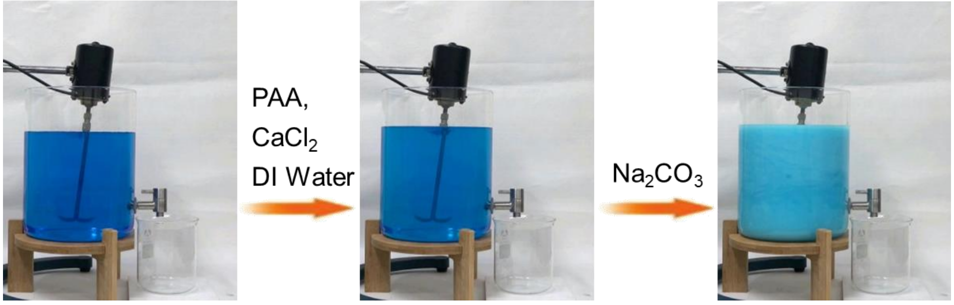

Figure S3. Purification of a multi-component solution without GO.

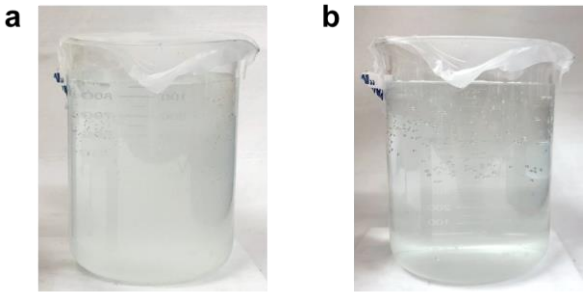

Figure S4. (a) As obtained decolorized filtrate. (b) Clarified filtrate after standing.

Table S1. Comparative experiments on precipitation of MB.

Materials	V_{MB} (9.5 mg·L^{-1}	V_{PAA} (0.4 M)	V_{CaCl_2} (0.4 M)	V_{GO} (1 mg·mL^{-1})	$V_{DI\ water}$	$V_{Na_2CO_3}$ (0.1 M
Experiment Group	10 mL	10 mL	10 mL	10 mL	0	10 mL
Control Group	10 mL	10 mL	10 mL	0	10 mL	10 mL
Blank Group	10 mL	0	0	0	40 mL	0

Table S2. Comparative experiments on precipitation of heavy metal ions.

Materials	$V_{PbCl_2/CdCl_2}$ (100 mg·L^{-1}	V_{PAA} (0.4 M)	V_{CaCl_2} (0.4 M	V_{GO} (1 mg·mL^{-1}	$V_{DI\ water}$	$V_{Na_2CO_3}$ (0.1 M
Experiment Group	10 mL	10 mL	10 mL	10 mL	0	10 mL
Control Group	10 mL	10 mL	10 mL	0	10 mL	10 mL
Blank Group	10 mL	0	0	0	40 mL	0

Table S3. Comparative experiments on precipitation of TiO_2.

Materials	V_{TiO_2} (250 mg·L^{-1})	V_{PAA} (0.4 M)	V_{CaCl_2} (0.4 M)	V_{GO} (1 mg·mL^{-1})	$V_{DI\ water}$	$V_{Na_2CO_3}$ (0.1 M)
Experiment Group	10 mL	10 mL	10 mL	10 mL	0	10 mL
Blank Group	10 mL	0	0	0	40 ml	0

Table S4. Comparative experiments on the removal of MB by precipitation.

Materials	V_{TiO_2} (250 mg·L^{-1})	V_{PAA} (0.4 M)	V_{CaCl_2} (0.4 M)	V_{GO} (1 mg·mL^{-1})	$V_{DI\ water}$	$V_{Na_2CO_3}$ (0.1 M)
Experiment Group	10 mL	10 mL	10 mL	10 mL	0	10 mL
Control Group	0	10 mL	10 mL	10 mL	10 mL	10 mL

Table S5. Comparative experiments on purification of large-scale, multi-component complex solutions.

Materials	Experiment Group - 1st	Experiment Group - 2nd	Control Group	Blank Group
$V_{MB\ (6\ \mathrm{mg}\cdot L^{-1})}$	4000 mL	4000 mL	4000 mL	4000 mL
$V_{CdCl_2\ (1\ \mathrm{g}\cdot L^{-1})}$	500 mL		500 mL	500 mL
$V_{PbCl_2\ (0.5\ \mathrm{g}\cdot L^{-1})}$	500 mL		500 mL	500 mL
$V_{TiO_2\ (350\ \mathrm{mg}\cdot L^{-1})}$	500 mL		500 mL	500 mL
$V_{GO\ (1\ \mathrm{mg}\cdot \mathrm{m}L^{-1})}$	200 mL	30 mL	0	0
$V_{PAA\ (2\ M)}$	200 mL	30 mL	200 mL	0
$V_{CaCl_2\ (2\ M)}$	100 mL	15 mL	100 mL	0
$V_{DI\ water}$	0	0	200 mL	600 mL
$V_{Na_2CO_3\ (1\ M)}$	100 mL	15 mL	100 mL	0

Table S6. Comparative experiments of control group on the removal of MB by precipitation.

Materials	Control Group
$V_{MB\ (6\ \mathrm{mg}\cdot L^{-1})}$	4000 mL
$V_{CdCl_2\ (1\ \mathrm{g}\cdot L^{-1})}$	500 mL
$V_{PbCl_2\ (0.5\ \mathrm{g}\cdot L^{-1})}$	500 mL
$V_{TiO_2\ (350\ \mathrm{mg}\cdot L^{-1})}$	0
$V_{GO\ (1\ \mathrm{mg}\cdot \mathrm{m}L^{-1})}$	200 mL
$V_{PAA\ (2\ M)}$	200 mL
$V_{CaCl_2\ (2\ M)}$	100 mL
$V_{DI\ water}$	500 mL
$V_{Na_2CO_3\ (1\ M)}$	100 mL
